# Implementation of antimicrobial resistance surveillance in Ghana using the Integrated Disease Surveillance and Response strategy

**DOI:** 10.4102/ajlm.v13i1.2404

**Published:** 2024-08-13

**Authors:** Beverly Egyir, Alfred Bortey, Kwabena O. Duedu, Gifty Boateng, Franklin A. Bekoe, George Hedidor, Michael Adjabeng, Nicholas T.K.D. Dayie, Noah Obeng-Nkrumah, Japheth A. Opintan

**Affiliations:** 1 Department of Bacteriology, Noguchi Memorial Institute for Medical Research, Accra, Ghana; 2 College of Life Sciences, Birmingham City University, Birmingham, United Kingdom; 3 National Public Health and Reference Lab, Korle-Bu Teaching Hospital, Korle-Bu, Ghana; 4 Disease Surveillance Unit, Ghana Health Service, Accra, Ghana; 5 World Health Organization Country Office, Ghana, Accra, Ghana; 6 Department of Medical Microbiology, University of Ghana Medical School, Accra, Ghana; 7 Department of Medical Laboratory Sciences, School of Biomedical and Allied Health Sciences, Accra, Ghana

## Introduction

The Integrated Disease Surveillance and Response (IDSR) strategy is aimed at enhancing public health surveillance and response in the African region by establishing connections across community, health facility, sub-district, district, regional, and national levels.^1^ The technical guidelines for IDSR were originally developed by the World Health Organization (WHO) Regional Office for Africa, and the United States Centers for Disease Control and Prevention for the broader African context.^2,3^ The IDSR strategy optimises resource utilisation by consolidating and streamlining common surveillance activities, including detection, reporting, analysis, interpretation, feedback, and action. This approach leverages shared structures, processes, and personnel to enhance efficiency in public health surveillance and response. The strategy also integrates the One Health perspective, addressing events that intersect human, domestic animal, wildlife, and the environment.^4^

The IDSR approach was adapted by Ghana to provide public health personnel, managers and decision-makers with relevant, adequate and timely data that helps in determining disease burdens, ensuring proper allocation of resources for early detection of outbreaks and to provide rapid public health response. Ideally, the strategy should also include monitoring of antimicrobial susceptibilities to promote efficient use of information for decision-making to combat antimicrobial resistance (AMR). It’s worth noting that systematic monitoring of antimicrobial susceptibilities of bacterial species is not integrated into the existing public health structures with the IDSR strategy in Ghana. This is due to challenges related to logistics, infrastructure and human resource support to ensure the comprehensive implementation of AMR surveillance by laboratories within the network of a sentinel surveillance system using the IDSR approach.

Antimicrobial resistance is an important global health threat and considered a public health emergency that requires attention. In response to this threat, countries have developed action plans and instituted measures to reduce its impact. Considering the huge clinical and economic impact caused by AMR pathogens, it is imperative to purposively implement AMR surveillance for a more holistic integrated surveillance system to combat AMR and infectious diseases.

In this article, we advocate for and offer recommendations regarding the implementation of AMR surveillance within the IDSR framework to facilitate real-time monitoring of AMR pathogens as well as the timely identification of threats posed by AMR pathogens to critical antimicrobials.

## The need: Purposeful implementation of antimicrobial resistance surveillance using the Integrated Disease Surveillance and Response approach

Over the past 20 years, 94% (44/47) of the countries in the WHO African region have been using the IDSR approach to intensify surveillance for specified priority diseases and conditions which have been met with great improvements.^5,6,7^ As at 2020, the data available show how the IDSR approach aided in the identification, investigation and response to outbreaks of measles, yellow fever, hepatitis E virus, circulating vaccine-derived poliovirus type 2, and coronavirus disease 2019 in many countries.^8^

Over time, there has been an increase in the resistance of bacteria to various antimicrobials.^9^ According to Murray et al., about 4.95 million deaths globally were associated with AMR, where 1.27m deaths were specifically attributed to AMR in 2019.^10^ Western sub-Saharan Africa recorded the highest mortality rates globally (27.3 deaths per 100 000) attributable to AMR, which shows that it is a matter of great concern in the sub-region.^10^

Mortality rates related to AMR in the sub-Saharan African region result from various factors, including ineffective infection prevention and control practices, inadequate management of healthcare systems, and insufficient data on the AMR burden in the region. Therefore, it is prudent and crucial to deliberately implement AMR surveillance through the IDSR to address the AMR challenge in sub-Saharan African countries. To a large extent, several healthcare facilities in Ghana, Tanzania and Zambia do not adequately use the IDSR technical guidelines for identification of cases in laboratories, and are not properly equipped for identification and confirmation of pathogens for priority diseases/conditions, including antimicrobial susceptibility testing of bacteria species.^11,12,13^

Turner et al. noted that high-quality surveillance is essential to cover the major themes of the specific objectives of the WHO Global Antimicrobial Resistance Surveillance System, which include the estimation of AMR burden, trends over time, and the impact of interventions.^8,14^ Ideally, an AMR surveillance system must have both clinical and laboratory data, and fortunately, the IDSR case-based surveillance system of Ghana collects clinical and laboratory data. However, the existing laboratory tests conducted are just to confirm the suspected diseases and not necessarily to keep track of pathogens and AMR profiles of bacteria species in the communities or regions.^15^ Exceptional steps are taken in this direction during meningitis outbreaks, where there is constant monitoring of the susceptibility of meningococcal bacteria causative agent to available antibiotics to inform case management.

In clinical settings, data from AMR surveillance are essential to inform empiric treatments.^14,16^ The selection of appropriate antimicrobial therapy has the potential to reduce the further rise and spread of AMR as well as the rate of healthcare-associated infections and multi drug-resistant organisms.

## The Integrated Disease Surveillance and Response framework and antimicrobial resistance surveillance

The IDSR strategy involves the collection of health-associated data using standardised tools via two major methods: the Indicator Based Surveillance and Event Based Surveillance.^4^ The Indicator Based Surveillance channel focuses on identification, collection, monitoring, analysis, and interpretation of structured data from health-based formal sources. On the other hand, the Event Based Surveillance is designed for rapid capture of information about events that are of potential risk to public health. It starts as an alert which is triggered based on an early warning and response system.

**FIGURE 1:**
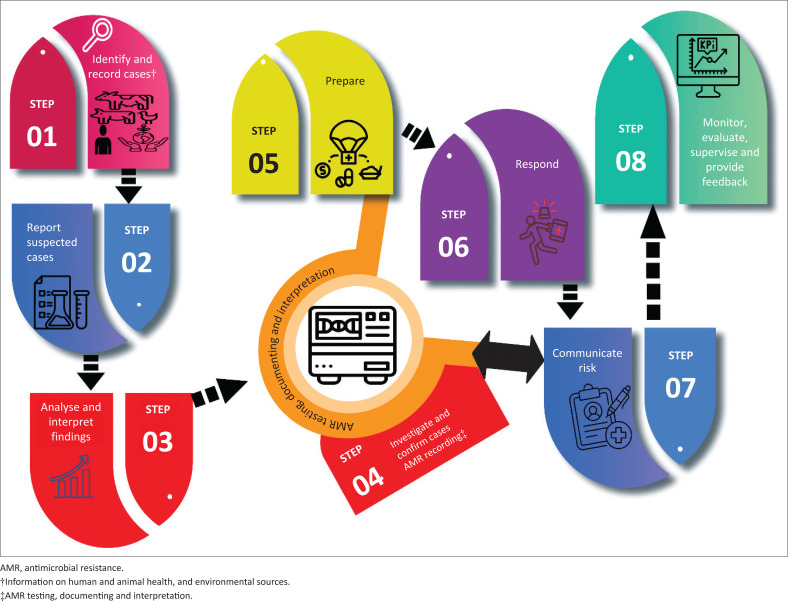
Diagram showing a suggested workflow incorporating antimicrobial resistance surveillance into the Integrated Disease Surveillance and Response matrix, using the One Health approach.

The existing IDSR matrix comprises eight vital core functions and activities that are to be performed by health system levels (Figure 1).^2^ Step 1 includes identifying and recording cases, conditions, and events using standard and simplified case definitions at health facility and community levels. Step 2 entails reporting of suspected cases and any potential Public Health Emergency of International Concern, conducting investigations, and submitting detailed reports. Step 3 involves analysing and interpreting surveillance data based on person, place, and time for public health actions. Step 4 entails investigating and confirming suspected cases or outbreaks through epidemiological investigation and laboratory testing. Hereafter, organisms detected from suspected samples should be recorded and reported on the priority diseases. The distributions and prevalence should be analysed and communicated with experts and officials (this serves as interlink between step 4 and step 7). Step 5 involves preparing by having emergency response plans, stockpiling essential supplies, and training personnel in advance. Step 6 involves responding to outbreaks by determining the cause, mobilising resources, and establishing coordination mechanisms. Step 7 entails engaging in risk communication with experts, officials, and at-risk communities to exchange information and advice. Step 8 includes monitoring, evaluating, and providing feedback to improve the surveillance system’s effectiveness, involving community representatives, the private sector, and non-governmental organisations in evaluation activities.

Considering the gravity of the AMR threat, we advocate for the implementation of AMR surveillance in the IDSR framework via the Indicator Based Surveillance and Event Based Surveillance channels on a sentinel basis. The Indicator Based Surveillance component can include defining country-specific priority pathogens and antimicrobials, among others. Ghana, as a signatory to the United Nations and the WHO, will naturally defer to the WHO-defined priority pathogens and antimicrobials. Further to this, we recommend that the country uses its existing surveillance structures and research data to conduct an assessment, and to identify any other pathogen or antimicrobial agent that is of importance to be included into Ghana’s list of priority pathogens and antimicrobials.

Ghana already has an electronic digital disease surveillance platform in use; the existing case-based reporting platform is already integrated into the Surveillance Outbreak Response Management and Analysis System. The case-based forms for AMR surveillance can be integrated into the Surveillance Outbreak Response Management and Analysis System platform which has already received significant investment for its deployment and training of health personnel to use it. During the coronavirus disease 2019 pandemic, the use of the Surveillance Outbreak Response Management and Analysis System reduced the reporting time lapse from result generation in the lab to the consulting room. It also provided real-time updates for public health responses. We predict that the data on antimicrobials and pathogens will be huge; hence, an artificial intelligence-driven algorithm will be needed to analyse data in real time to identify patterns of concern which will be sent to designated public health officials to review and report for response action. The type of feedback from the artificial intelligence algorithm should allow classification of threats. In response to the indicator data, response plans can be developed and deployed.

The Event Based Surveillance, on the other hand, will encompass swift collection of data related to events that may pose a potential risk to public health. It commences as an alert, triggered by an informal and not well-defined early warning and response system.

## Integrated Disease Surveillance and Response and microbiology facilities in Ghana

**FIGURE 2:**
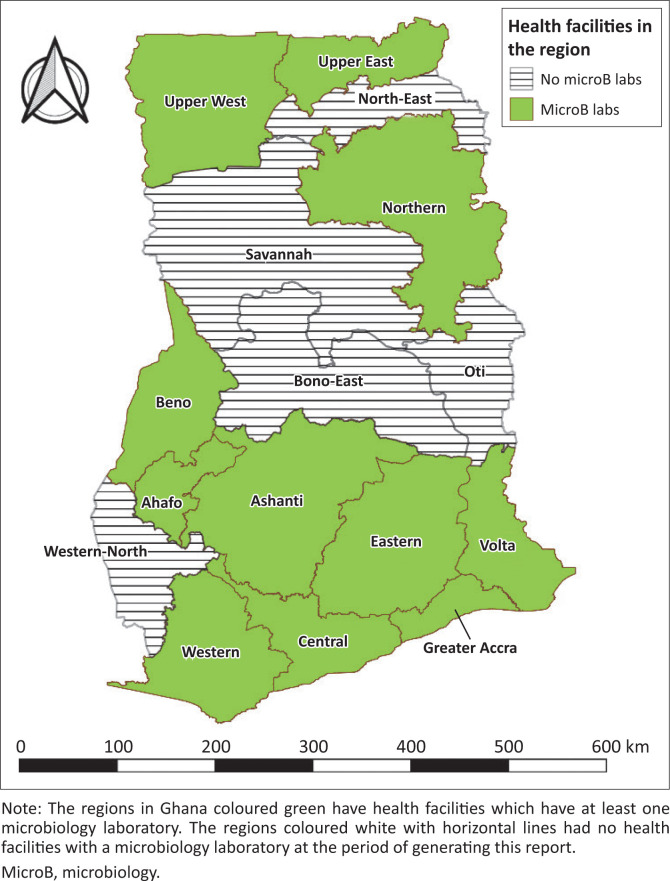
Map of Ghana showing microbiology laboratories involved in Integrated Disease Surveillance and Response testing of priority conditions in Ghana, May 2024.

According to the IDSR framework, health facilities are supposed to identify suspected cases of diseases, notify, confirm them through laboratory testing and report on them as well. However, there are districts in Ghana with neither hospitals nor microbiology laboratories. The absence of such needful capacity is a great limitation to the nationwide success of AMR surveillance. Out of 32 health facilities sampled in the Ga West Municipality, only six facilities had IDSR guidelines in their facilities, while nine out of 32 submit weekly reports, and 19 submit monthly reports.^17^ Such information highlights the need for improvement and capacity building of laboratories at health facilities in Ghana to promote accurate, efficient, and effective disease and AMR surveillance in Ghana. Figure 2 shows microbiology laboratories involved in testing of priority conditions in Ghana.

## Perceived hindrances and recommendations

In April 2018, Ghana introduced the Antimicrobial Use and Resistance Policy and National Action Plan on AMR, aligning with the objectives of the Global Action Plan on AMR.^18^

Ghana has established national platforms, including the National AMR Platform and the National One Health Platform, which comprise representatives from the human, animal, and environmental sectors. These platforms continuously deliberate on AMR, One Health, and related issues to address public health impacts.

To enhance understanding of AMR through surveillance and research, Ghana secured the initial Fleming Country Grant. This grant supported a government-led initiative to collect, analyse, and report AMR and antimicrobial use data from humans and animals across 11 sentinel sites. The goal was to generate a comprehensive national AMR overview to guide treatment decisions at both local and national levels. Extensive engagement, training of laboratory staff, and preparation for a pilot surveillance were carried out at 11 sentinel sites. The sentinel sites or clinical microbiology laboratories received infrastructure upgrades with assistance from the Fleming Fund. This successful approach can be expanded and sustained within the framework of the IDSR for AMR surveillance in the country.

It is worth noting that implementing effective or sustainable AMR surveillance programmes in Africa, including Ghana, comes with a plethora of challenges. These could range from the lack of resources (material and human), improper or inadequate infrastructure, poor supply chain for laboratory consumables and inadequate data generation, collation, analysis and sharing. Also, in places where there are laboratory facilities that can help in AMR surveillance, health workers are not motivated enough to go beyond their normal duties due to the constant work overload in their facilities.

Some facilities in Ghana can produce antibiograms from their laboratories, but the lack of sufficient biodata hinders the comprehensive assessment and analysis of the AMR data. This calls for continuous training of health personnel and laboratory staff on data management, and the importance of surveillance. Training programmes should cover all categories of personnel within the health delivery system, extending from the local to the regional and national levels.

Facilities lacking sufficient capacity to confirm the identity of pathogens can collaborate with national reference laboratories. In all the 16 regions of Ghana, there are 11 regional or designated hospitals with microbiological labs. Facilities without the relevant infrastructure can send isolates, along with appropriate epidemiological and clinical data to reference labs, for the identification and susceptibility testing of these pathogens. The reference laboratories must provide feedback to the submitting facilities regarding AMR patterns of the pathogens, and where sequencing instruments are available in reference laboratories, whole genome sequencing can also be performed on the pathogens to generate granular data to inform surveillance activities as well as monitor the rise and spread of epidemic and pandemic clones. With support from the Fleming Fund’s regional grant, the SeqAfrica consortium is offering whole genome sequencing and data analysis services in Ghana (and other African countries). This initiative has led to the sequencing of several AMR bacterial species from the environment, animals, and humans to strengthen AMR surveillance in the country.

Antibiogram information should be generated at the local, regional, and national levels to guide empirical antibiotic prescribing in hospitals. For example, a 6-month antibiogram data plot generated from clinical microbiology laboratories in hospitals can be pasted in consulting rooms to inform antimicrobial choices for patients. Information on the overall microbiological cultures performed, the number of positive cultures, prevalence of AMR pathogens, and whether the infections are community or hospital acquired, are additional information (currently not collected), and can be obtained as part of the core indicators of the existing IDSR framework.

Antimicrobial susceptibility testing data from private hospitals and laboratories can also be harnessed within the IDSR framework to gain a deeper understanding of the AMR profiles of bacterial pathogens in the country.

## Conclusion

Implementing AMR surveillance within IDSR strategy is a pivotal move towards identifying AMR pathogens, particularly those implicated in outbreaks. Furthermore, this implementation aligns with global health initiatives, reinforcing Ghana’s commitment to combating AMR and infectious diseases. By providing timely and accurate data on AMR trends, the IDSR framework will facilitate informed decision-making in healthcare policies, resource allocation, and public health interventions. Ultimately, this endeavour will significantly contribute to safeguarding public health, improving patient outcomes, and curbing the spread of AMR pathogens, marking a major stride in Ghana’s ongoing battle against AMR and infectious diseases.
